# Synthesis and electrochemical properties of nanocubes Mn_2_SnS_3_ for high-performance supercapacitors

**DOI:** 10.1038/s41598-023-47738-w

**Published:** 2023-11-27

**Authors:** Mona Sanayee, Majid Arvand

**Affiliations:** Electroanalytical Chemistry Laboratory, Faculty of Chemistry, University of Guilan, Namjoo Street, P.O. Box: 1914–41335, Rasht, Iran

**Keywords:** Chemistry, Energy science and technology, Materials science, Nanoscience and technology

## Abstract

Exploring environment-friendly active material-electrolyte combinations has become increasingly necessary with the rising use of supercapacitors. In this study, the potential of ternary Mn_2_SnS_3_ on Ni foam as an electrode material was considered. The study investigated the impact of precursors on the morphology of the prepared electrodes utilizing techniques such as X-ray diffraction, energy dispersive X-ray analysis, field-emission scanning electron microscopy, and transmission electron microscopy. Nanocubes Mn_2_SnS_3_ (NC-MTS) and nanoworms Mn_2_SnS_3_ (NW-MTS) were synthesized via a facile solvothermal route. The results suggest that NC-MTS exhibits better capacitive performance compared with NW-MTS, which means that morphology has a significant effect on the electrochemical reaction. NC-MTS presents excellent supercapacitor performances with a high specific capacity of about 2115 F g^−1^ at current density 2 A g^−1^, excellent rate capability of 78% at 17 A g^−1^ and excellent cycling stability 92% capacitance retention after 3000 GCD cycles. Whereas, NW-MTS illustrated a specific capacity of about 853 F g^−1^ at current density 2 A g^−1^, rate capability of 50% at 17 A g^−1^ and cycling stability of 81% capacitance retention after 3000 GCD cycles. Additionally, an asymmetric supercapacitor NC-MTS/NF//AC based on the NC-MTS/NF as a positive electrode and activated carbon (AC) as a negative electrode was successfully constructed with the excellent electrochemical performance, which demonstrated a high energy density of 60.56 Wh kg^−1^ and a high power density of 699.89 W kg^−1^.

## Introduction

Some of the major challenges confronting the world include escalating fuel costs, higher energy consumption driven by economic growth, the threat of global warming, heightened pollution levels, and geothermal issues^1^. As a result of these issues, different research projects have been undertaken to explore alternative energy sources^2^. Renewable energy sources that can be used as a substitute for traditional fossil fuels are commonly known as alternative energy sources. A few examples of studies that have been conducted to develop alternative sources of energy include solar energy, wind energy, geothermal energy, hydrogen fuel, and biofuels. These are just a few examples of the many studies that have been conducted to develop alternative sources of energy. Accordingly, the storage of various types of energy became an important topic^3–5^. Overall, energy storage technologies are evaluated on various factors such as energy density, storage capacity, reliability, environmental sustainability, and durability^6–9^.

Researchers and engineers are mainly involved in two forms of electrochemical devices: batteries and capacitors, all of which are promising energy storage systems. Furthermore, depending on charge management mechanisms, all energy storage technologies have advantages and disadvantages. Batteries have a low power density but a high energy density, whereas traditional capacitors have a high power density but a low energy density. Supercapacitor (SCs), with significant benefits, such as increased strength and energy flow, long cycle life, flexible operating temperature, and environmental friendliness, are intermediaries between the battery and the traditional capacitor^10–13^. Supercapacitors can be broadly classified into three categories based on the mechanism used to store electrical charge: (I) The electrical double layer capacitor (EDLC), capacitance is attained in this system by the accumulation of purely electrostatic charge at the interface between the electrode and the electrolyte. The availability of electrode surface area to the electrolyte ions is a critical factor upon which this group heavily depends. This class of systems involves a physical process of charge separation that does not involve any Faradaic reactions occurring on the electrode surface^14^. (II) Pseudocapacitors, charge storage initiates from Faradic charge transfer at the interface. In essence, pseudocapacitance is a faradic reaction similar to the battery-like redox reaction; however, it takes place at a rate equivalent to those of EDLCs, which is reflected by its electrochemical behavior. This pseudocapacitive behavior can originate from different phenomena such as doping, redox, and intercalation. Like EDLCs, pseudocapacitors have been studied using other active materials and electrolytes to improve their performance under different working environments^15,16^. (III) The hybrid group combines the characteristics of both EDLCs and PSCs to create a unique energy storage system. PSCs have the potential to deliver higher charge storage capacities than EDLCs due to their ability to facilitate fast and reversible redox reactions^17^. Generally, the efficiency of supercapacitors can be significantly boosted by carefully selecting electrode materials, which requires a thorough understanding of the charge storage mechanisms, electrochemical conditions, and pathways for the transport of ions and electrons^18^.

In order to overcome the disadvantages of supercapacitors such as low density, low cell voltage, and fast discharge rates, researchers are actively searching for novel electrode materials. Generally, supercapacitors require electrode materials to possess high conductivity, porosity, and large surface area to realize a high specific capacitance (*C*_s_). Moreover, *C*_s_ depends on the choice of intrinsic behavior of the active material. Therefore, the selection of electrode material should satisfy several parameters, including high electronic conductivity to enable improved *C*_s_, nanostructure morphology, high surface area, controlled porosity, and high energy and power densities of the capacitor. For this reason, researchers are trying to develop new electrode materials for SCs^19^. Initially, carbon materials were used for supercapacitor fabrication because of their distinct characteristics, comprising of extensive surface area, great conductivity, and superior stability^20^. However, in recent years, other materials such as transition metal oxides and conducting polymers have been considered for use in supercapacitor^21^. Transition metal sulfides (TMSs) demonstrates unique electronic structures and physical properties because of their distinct geometric structures having weak interlayer van de Waals coupling, different compositions, and rich phase structures providing a library of materials for potential applications in energy storage and conversion devices in comparison to their corresponding metal oxides^22,23^. In addition, compared to monometallic sulfides, ternary transition metal sulfides exhibit a greater range of oxidation states, which leads to more extensive redox reactions and ultimately results in higher specific capacitance^24^. Additionally, the substitution of sulfur for oxygen led to increased flexibility resulting from sulfur's lower electronegativity^25^, hindering structural changes by elongation between layers and providing a short transport pathway for ions and electrons^26^. The lower band gap in TMSs results in higher electron conductivity than their corresponding oxides, making them a highly desirable choice for supercapacitor applications^27^. Consequently, much attention has been directed towards designing and fabricating TMSs for supercapacitors due to their availability, eco-friendly nature, high conductivity, high stability, and excellent electrochemical performance compared to single metal oxides^28^. Alipour et al.^29^ synthesized MnMoS_4_ nanosheets on nickel foam as a supercapacitor via two-step hydrothermal method. The specific capacitance reached 1865.2 F g^−1^ at a current density of 1 A g^−1^. Zhao et al.^30^ designed pinecone-like and hierarchical manganese cobalt sulfide, which delivered a specific capacitance of 992 F g^−1^ at a current density of 1 A g^−1^. Yang et al.^31^ synthesized hollow carbon spheres using yeast cells (YC) as the carbon source. NiCo_2_S_4_ nanosheets were further grown on the YC surface by hydrothermal synthesis. The YC/NiCo_2_S_4_ composite exhibited great electrochemical performance (specific capacitance of 747 F g^−1^ at 1 A g^−1^). Ning et al.^32^ synthesized nitrogen-doped carbon nanofibers derived from bacterial cellulose (CBC-N) as a template for hydrothermal growth of NiCo_2_S_4_ in a confined space and as a conductive negative electrode material for constructing an asymmetric supercapacitor. The CBC-N@NiCo_2_S_4_ composite exhibited a high capacitance of 1078 F g^−1^ at 1 A g^−1^. Dai et al.^28^ synthesized CoMoS_4_ nanoparticles via a facile chemical co-precipitation process, which delivered a specific capacitance of 395 F g^−1^ at a current density of 1 A g^−1^. Among the metal sulfides, stannous sulfide (SnS) is an appealing electrode material for electrochemical capacitors. SnS is increasingly notable owing to its particularly semiconducting properties^33^. Due to their layered structure that enables the intercalation of ions during electrochemical reactions, tin sulfides have garnered attention as a promising alternative material for use as an anode in supercapacitor applications^34^. In addition, they have high chemical stability, and both Sn and S exhibit low toxicity and are not harmful to the environment. Their non-toxicity and abundance in nature make them ideal materials for the fabrication of ecologically safe PSCs^35^. Despite their potential for use in supercapacitors, the lower specific capacitance of tin sulfides has limited their application. This is partly due to their poorer electrical conductivity compared to other metal sulfides. To address this limitation, the addition of transition metals such as Cu and Mn to SnS can enhance its electrical conductivity and improve its electrochemical performance^36^. In this context, the ternary compound manganese tin sulfide (Mn_2_SnS_3_) is a promising material for various applications due to its non-toxicity, high abundance of constituent elements, excellent electrochemical performance and environmental stability. Mn_2_SnS_3_ also demonstrates p-type electrical conductivity and features a high optical absorption coefficient (> 104 cm^−1^), and its band gap can be modulated (0.9–1.77 eV) by altering its structure^37^. Thus, Mn_2_SnS_3_, a ternary semiconductor compound, has emerged as a promising candidate for use in supercapacitor electrode materials. Different methods have been utilized for the synthesis of Mn_2_SnS_3_ nanostructures, such as solvothermal/hydrothermal methods^38^, chemical bath deposition-sulfurization^39^, spin coating-sulfurization^40^, and drop-casting-sulfurization^41^. Regarding this matter, the solvothermal route is an appealing technique to produce desired morphologies with high selectivity, which can be advantageous for energy storage applications and improving the electrical characteristics of the material. In this investigation, diverse morphologies of Mn_2_SnS_3_ samples were synthesized via the one-step solvothermal method and evaluated as potential electrode materials for high-performance PSCs. The selection of solvent type, temperature, and reaction time were critical and effective parameters investigated in this research, as they impact the morphology and electrochemical performance of the electrode. Electrochemical performances of the Mn_2_SnS_3_ samples were evaluated in a standard three-electrode system, with the Ni foam coated with Mn_2_SnS_3_ serving as the working electrode. The Mn_2_SnS_3_ nanocubes (NC-MTS) exhibited a large gravimetric capacitance of 2115 F g^−1^ at a current density of 2 A g^−1^ and 91% preservation of initial capacity after 3000 cycles, while the Mn_2_SnS_3_ nanoworms (NW-MTS) revealed a gravimetric capacitance of 853 F g^−1^ at a current density of 2 A g^−1^ and 81% preservation of initial capacity after 3000 cycles. The Mn_2_SnS_3_/NF//AC asymmetric electrochemical capacitor device produced a high energy density of 60.56 Wh kg^−1^ and a high power density of 699.89 W kg^−1^, making it a promising candidate for energy storage systems. To demonstrate its capability as a power source, the device was employed to power light-emitting diodes (LEDs).

## Methods

### Synthesis of Mn_2_SnS_3_ nanocubes

MnCl_2**·**_4H_2_O, SnCl_2_·2H_2_O, SnCl_4**·**_5H_2_O, Na_2_S·9H_2_O, thiourea (TU), carbon black (CB), polytetrafluoroethylene (PTFE), and N-methyl-2-pyrrolidinone (NMP, C_5_H_9_NO) were obtained from Merck (Darmstadt, Germany) and used without further purification. Prior to the experiments, the Ni foam (0.5 × 0.5 cm^2^) underwent an ultrasonic cleaning process with 3 mol L^−1^ HCl solution, ethanol, and deionized water for 30 min to eliminate the surface oxide layer, followed by drying at 60 °C in a drying oven for one hour, while all other chemicals and reagents were used without further purification. To synthesize Mn_2_SnS_3_ nanocubes (NC-MTS), the typical procedure involved dissolving 0.4 g of MnCl_2_·2H_2_O, 0.225 g of SnCl_2_·2H_2_O, and 0.225 g of TU in 15 mL of ethylene glycol (EG). The resulting solution was stirred for 2 h to ensure complete dispersion and dissolution. The solution was then transferred into a Teflon-lined stainless autoclave, sealed, and heated in an oven at 180 °C for 16 h without undergoing annealing. Afterwards, the autoclave cooled naturally; the sample was centrifuged and washed several times with deionized water and absolute ethanol, then dried in a vacuum oven at 60 °C for 12 h.

### Synthesis of Mn_2_SnS_3_ nanoworms

A similar synthetic process was employed for the production of nanoworm-like Mn_2_SnS_3_ (NW-MTS). Specifically, 0.4 g of MnCl_2_·2H_2_O, 0.225 g of SnCl_4_·5H_2_O, and 0.225 g of sodium sulfide (Na_2_S) were dissolved in a solution of 50% ethanol and 50% water (30 mL).The resulting mixture was then placed into a sealed autoclave and kept at 200 °C for duration of 6 h. After natural cooling of the autoclave, the sample was subjected to centrifugation and washed repeatedly with deionized water and absolute ethanol, and then dried in a vacuum oven at 60 °C for 12 h. This investigation highlights the critical role played by temperature, reaction time, and solvent in determining the morphology of the synthesized samples, such that even slight alterations to these parameters can significantly impact the resulting structures.

### Characterization methods

The morphology and structural properties of the synthesized samples were analyzed using a combination of field emission scanning electron microscopy (FESEM, Mira3 XMU from TESCAN Company), transmission electron microscopy (TEM, JEOL 2010), and energy dispersive X-ray (EDX) analysis. X-ray diffraction (XRD) analysis, employing Cu *K*_α_ radiation (λ = 0.15418 nm, at 40 kV and 30 mA) from a Rigaku Denki Co. Ltd., Japan, was utilized to determine the crystal structure of the synthesized products. The surface area and pore size distribution were analyzed by the Brunauer–Emmett–Teller (BET) and the Barret-Joyner-Halenda (BJH) methods, using a BELSORP MINI II (BEL. Japan Inc.) at 77.0 K.

### Electrochemical characterization

To investigate the pseudocapacitive properties of the fabricated electrode material, electrochemical measurements were performed on the as-synthesized electrode using a conventional three-electrode cell configuration, with 3 mol L^−1^ KOH serving as the electrolyte solution and the μAutolab PGSTAT 30 electrochemical analyzer (Ecochemie BV, Utrecht, the Netherlands) controlled by Nova 2.1 software. A standard three-electrode system was used to assess the electrochemical performance of the samples, with the Mn_2_SnS_3_-coated Ni foam serving as the working electrode, Ag/AgCl (sat. KCl) acting as the reference electrode, and a platinum wire serving as the counter electrode. To prepare the working electrode, a homogeneous slurry was created by mixing the electrode material, CB, and PTFE in a ratio of 7:2:1, followed by the addition of NMP and stirring for 2 h. The resulting slurry was then applied to both sides of the nickel foam and dried in a vacuum oven at 60 °C for 12 h. The electrochemical properties of the samples were characterized using various techniques, including cyclic voltammetry (CV) over a potential window of 0 to 0.5 V, galvanostatic charge–discharge (GCD) tests through chronopotentiometry in the voltage range of 0 to 0.4 V, and electrochemical impedance spectroscopy (EIS) measurements across a frequency range of 0.01 Hz to 100 kHz and an amplitude of 5 mV ac. All of these techniques were performed using the same instrument. Zview software was utilized to fit the equivalent circuit to the impedance spectra. The specific capacitance of the electrodes in three-electrode mode was then determined by analyzing the GCD curves, using the following equation:

1 $$C\mathrm{sp} =\frac{I \times t}{\Delta V\times m}$$

where *C*_sp_ is the specific capacitance (F g^−1^ or F cm^−2^), *I* is the discharge current (A),* t* is the discharge time (s), Δ*V* is the potential window (V), and *m* is the mass of the active material (g). The energy density and power density of the electrodes were calculated from the following equations:

2 $$ED=\frac{{C}_{\mathrm{sp}}\Delta {V}^{2}}{2}$$

3 $$PD=\frac{E}{t}$$

where *ED* is the energy density (Wh kg^−1^), *C*_sp_ is the specific capacitance (F g^−1^), Δ*V* is the potential range (V), *PD* is the power density (W kg^−1^) and *t* is the discharge time (s).

### Fabrication of asymmetric supercapacitor (ASC)

For the fabrication of the asymmetric supercapacitor, a Mn_2_SnS_3_/NF electrode was utilized as the positive electrode, while activated carbon (AC) served as the negative electrode. The negative electrode was synthesized by applying a mixture of AC, CB, and PTFE binder (in a weight ratio of 8:1:1), dissolved in NMP, onto a nickel foam current collector and subsequently pressing and drying the electrode overnight at 60 °C. To assess the performance of the resulting Mn_2_SnS_3_/NF//AC hybrid device, a two-electrode setup was employed using 2 mol L^−1^ KOH as the electrolyte, with each electrode having a surface area of 0.5 cm^2^ and being immersed in an aqueous solution of KOH electrolyte. The potential range of the Mn_2_SnS_3_ cathode was from 0.0 to 0.5 V versus Ag/AgCl, while that of the AC anode electrode was 1.0–0.0 V versus Ag/AgCl, resulting in a total potential range of 0.0–1.5 V for the asymmetric Mn_2_SnS_3_/NF//AC cell. To ensure balanced charges stored on both electrodes (*Q*), the ratio of mass loading of electroactive materials on the positive and negative electrodes was determined using the following equation:

4 $${Q}_{+}={Q}_{-}\to {m}_{+}\times {\Delta V}_{+}\times {C}_{+}={m}_{-}\times {\Delta V}_{-}\times {C}_{-}$$

which *C* (F g^−1^) is specific capacitance, Δ*V* (V) is the potential range during the charge–discharge test, and *m* (g) is the mass of active material (the subscripts “ + ” and “–” refer to the positive and negative electrodes). The optimal weight ratio between the positive and negative electrodes was determined to be approximately *m*_+_/*m*_–_ ≈ 0.21. Furthermore, the specific energy and specific power of the ASC device were calculated using Eqs. (2) and (3), respectively.

## Results and discussion

### Fabrication and structural characterization of Mn_2_SnS_3_/NF electrode

**Figure 1:**
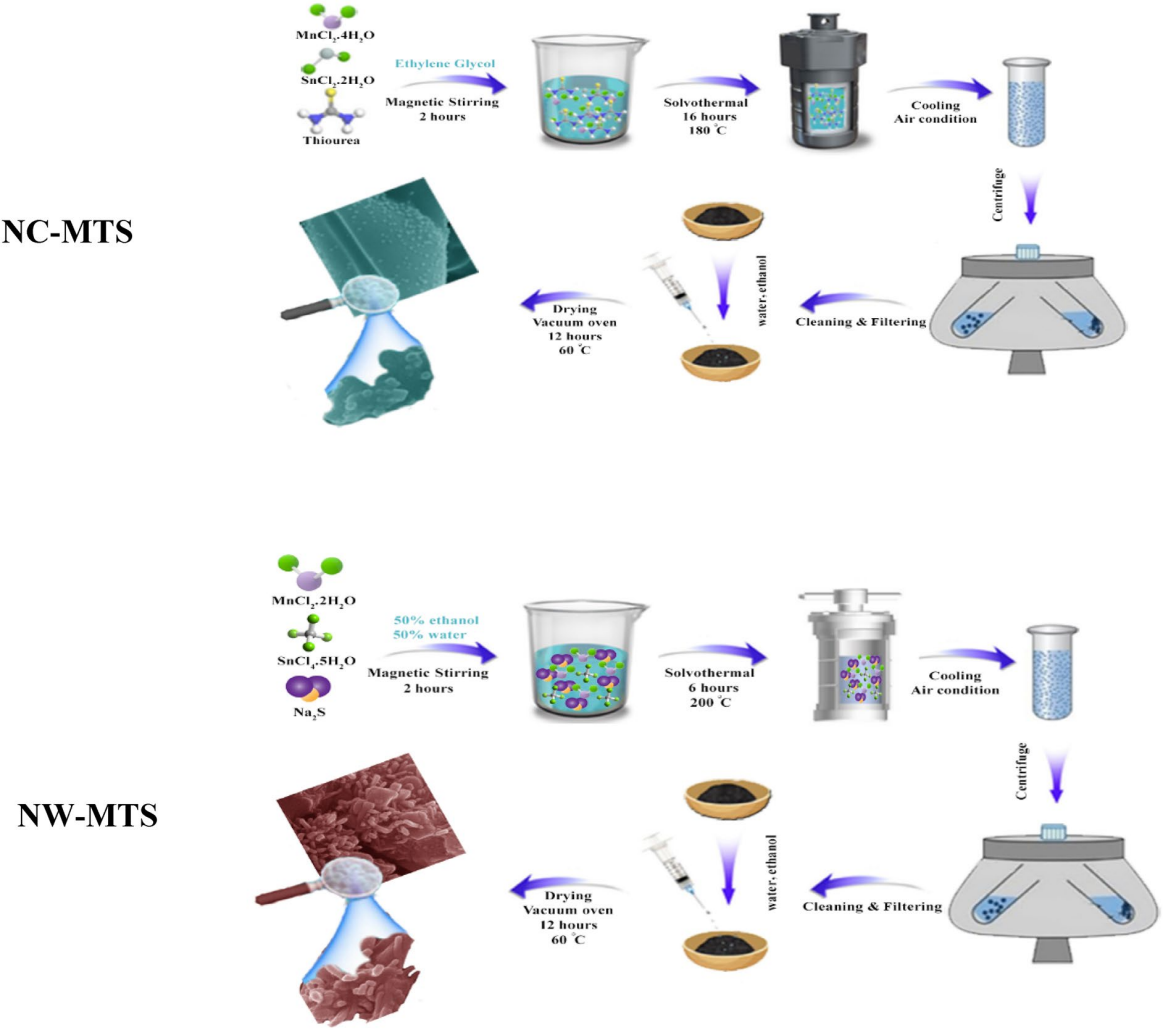
Schematic representation for the fabrication process of the Mn_2_SnS_3_/NF.

The synthesis process of Mn_2_SnS_3_/NF electrode is shown in Fig. 1. The process involved synthesizing Mn_2_SnS_3_ nanostructures with two distinct morphologies, using different precursors and temperature conditions. Once the successful synthesis of the desired morphologies was confirmed, the study investigated the effect of these morphologies on the electrochemical performance of the electrodes.

**Figure 2:**
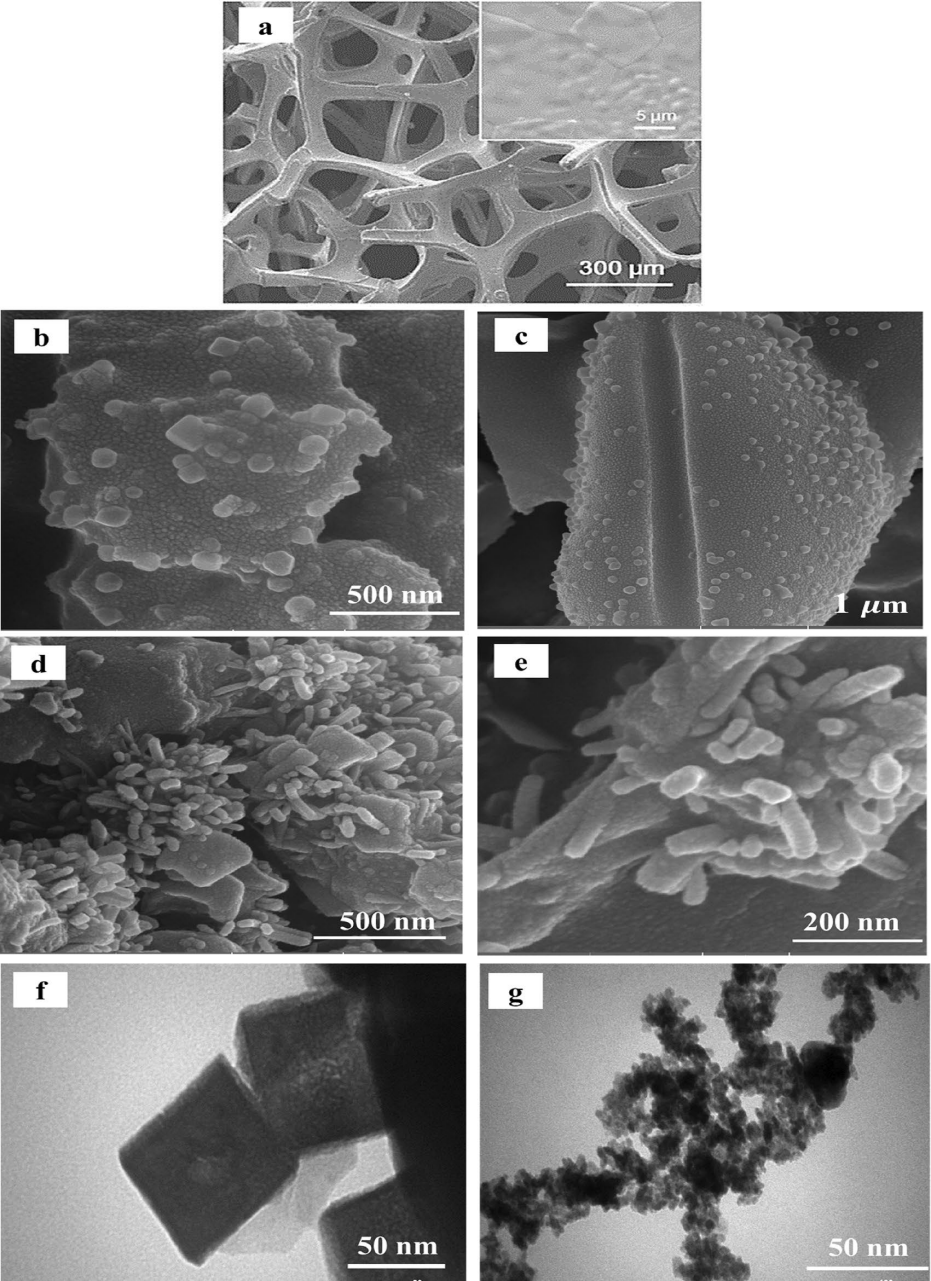
(**a**) FESEM image of the bare Ni foam; (**b**, **c**) high and low magnification FESEM images of NC-MTS on Ni foam; (**d**, **e**) FESEM images of NW-MTS on Ni foam; (**f**, **g**) TEM images of NC-MTS and NW-MTS respectively.

The morphologies of pristine Ni foam, NC-MTS and NW-MTS were characterized by FESEM. As shown in Fig. 2a, the commercial Ni foam has a 3D structure with a relatively plain surface. Ni foam was chosen as the substrate for its low cost, excellent conductivity, and uniform macroporous structure. This method offers a fast and effective route to enhance contact between the active material and the electrolyte, owing to its three-dimensional spatial configuration. Different morphologies of Mn_2_SnS_3_ are obtained by different synthetic conditions. The high and low magnification FESEM images of NC-MTS disclose that clear nanocubes with different sizes are arranged together on a porous texture, as illustrated in Fig. 2b,c. As well as, the high and low magnification FESEM images of NW-MTS were revealed by the FESEM images in Fig. 2d,e. It consists of numerous worm-like nanostructures stacked together on a porous network. Consequently, the resulting nanostructures possess a substantial surface area, allowing for efficient and widespread electrolyte access during electrochemical testing. Furthermore, TEM images were used for further investigations. Figure 2f,g display the typical TEM images of NC-MTS and NW-MTS. As can be seen, the structural characteristics of the synthesized materials were found to be consistent with the observations made from the FESEM images.

**Figure 3:**
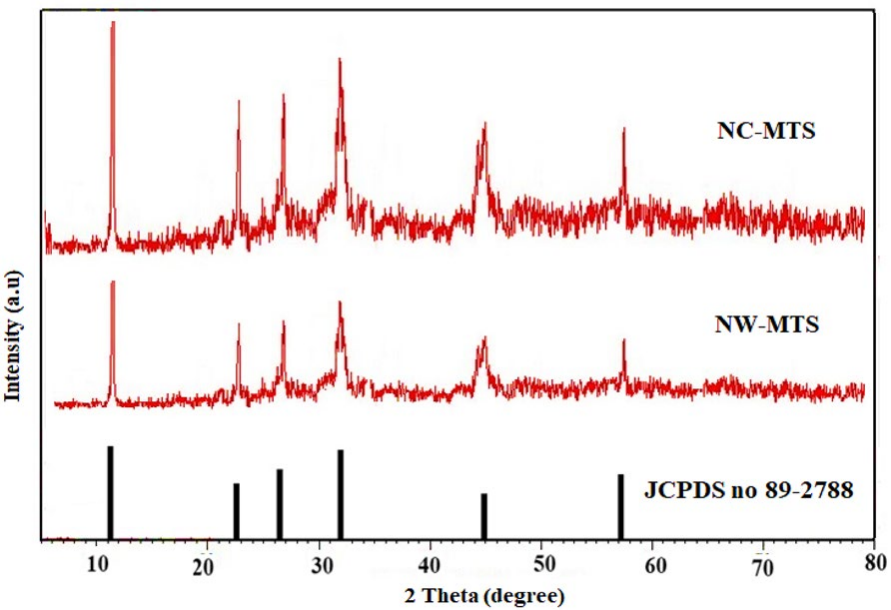
XRD patterns of as-prepared NC-MTS and NW-MTS.

The phase composition and crystallinity of Mn_2_SnS_3_ were verified by XRD patterns. Figure 3 illustrates the XRD patterns of NC-MTS and NW-MTS. As shown in Fig. 3, for Mn_2_SnS_3_ the major XRD diffraction peaks that evidenced at 2θ = 11.5°, 22.5°, 26.5°, 32°,45° and 57.5° correspond to (110), (111), (200), (211), (220), and (311) of Mn_2_SnS_3_ (JCPDS no. 89-2877), and no impurity diffraction peak is evident, which is in good agreement with the standard data for the cubic phase of the Mn_2_SnS_3_. Interestingly, the peak intensity of NC-MTS is higher than that of NW-MTS, implying that it has better crystallinity than that of NW-MTS.

**Figure 4:**
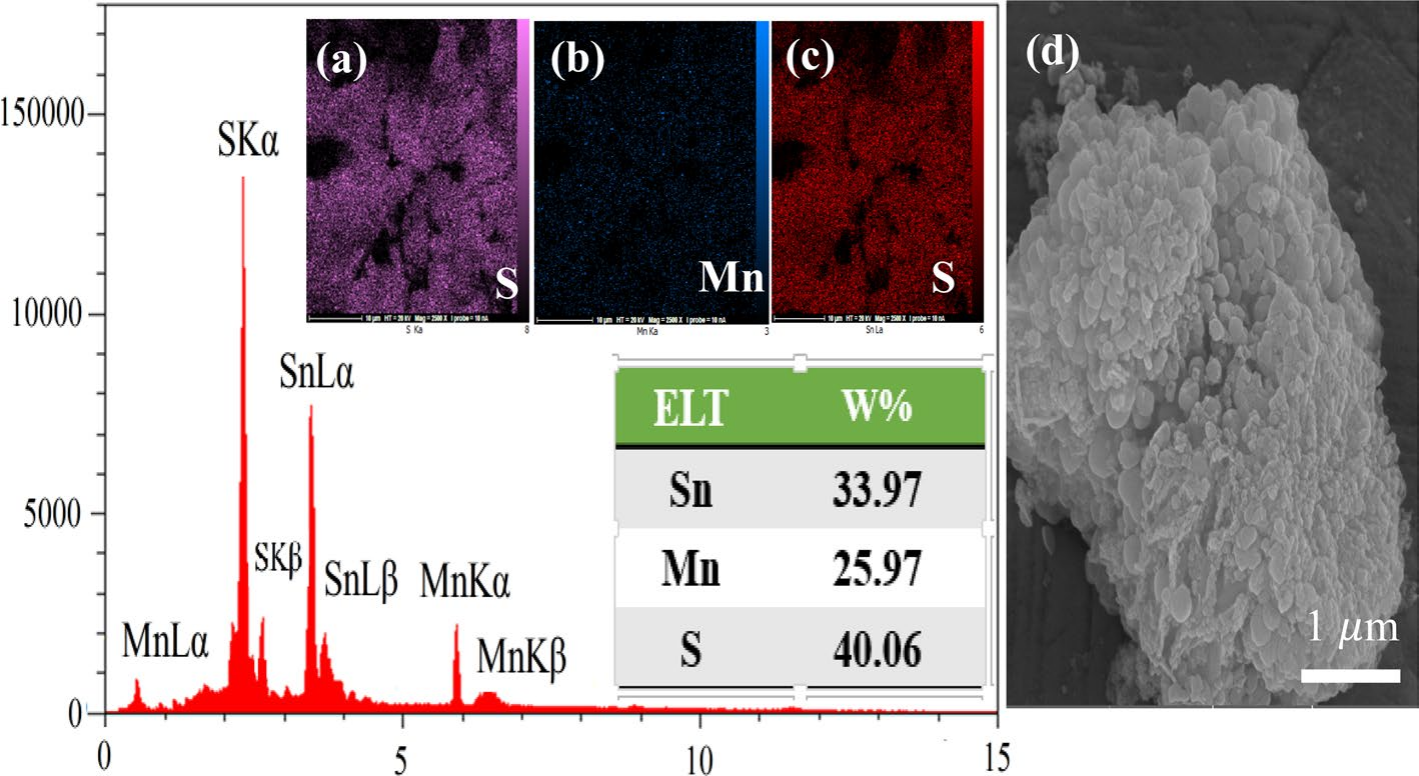
EDX spectrum; (**a**–**c**) the inset of elemental mapping; (**d**) original figure of SEM mapping of NC-MTS.

Moreover, the chemical composition of Mn_2_SnS_3_ was confirmed by energy-dispersive X-ray spectroscopy (EDX). As displayed in Fig. 4, the Mn_2_SnS_3_/NF consists of Sn, S and Mn elements, with percentages of 33.97%, 40.06% and 25.97%, respectively, which are uniformly distributed. The data obtained from the analysis suggests that the Mn_2_SnS_3_ nanostructures are of high purity, with little to no detectable impurities. Furthermore, the element mapping images of Mn_2_SnS_3_/NF in inset of Fig. 4 illustrates the uniform distribution of Sn, S, and Mn elements within the synthesized material.

**Figure 5:**
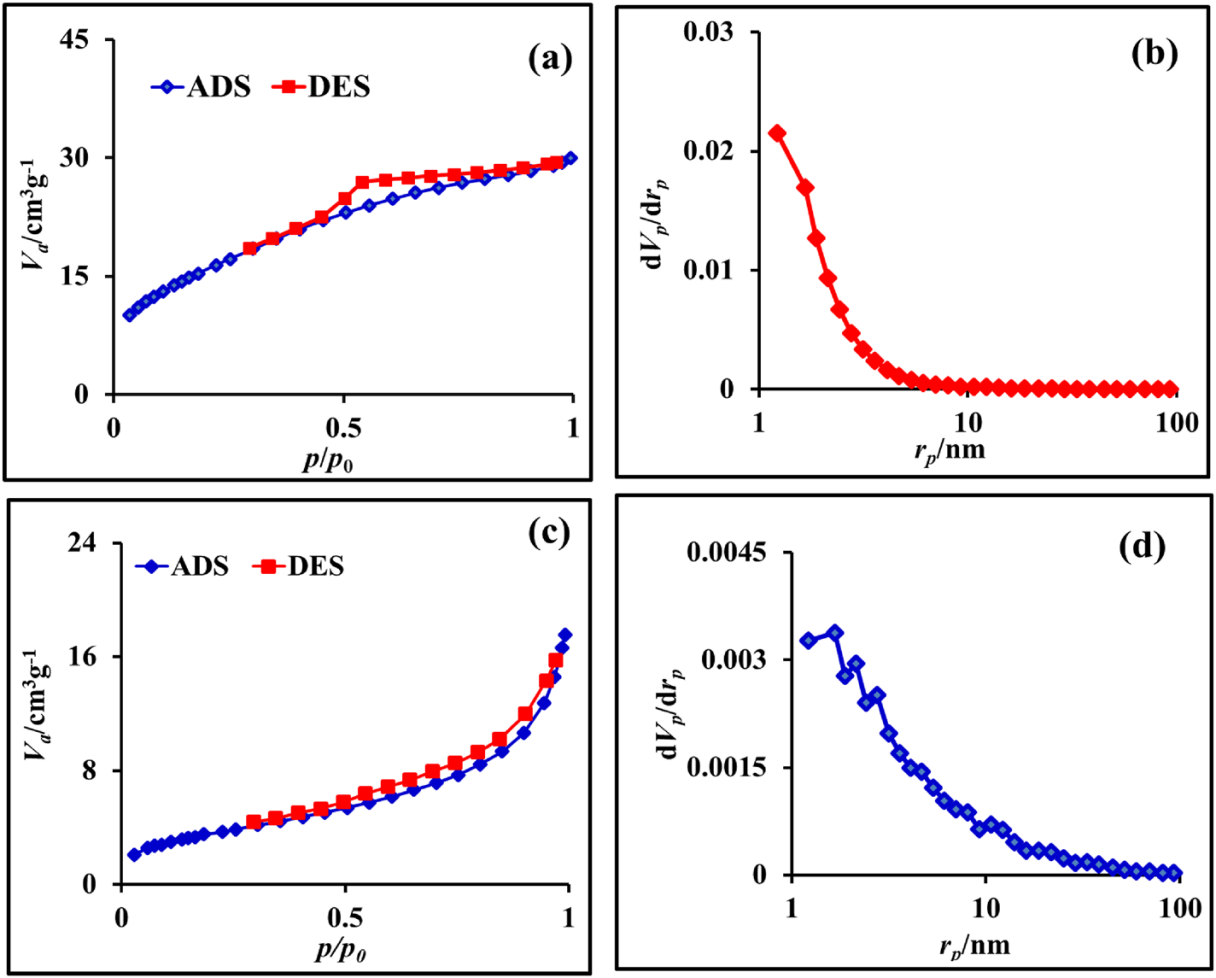
(**a**, **c**) Nitrogen adsorption–desorption isotherms; (**b**, **d**) pore size distribution curves of the NC-MTS and NW-MTS.

The specific surface area and porous structure of the as-prepared NC-MTS and the NW-MTS electrodes were also considered by the N_2_ adsorption/desorption analysis. Figure 5a and c demonstrate their nitrogen adsorption–desorption isotherms, and the specific surface areas were calculated by the BET method. BJH method was used to realize the pore volume and pore size distribution. The BET surface area value of the NC-MTS is estimated to be 52.803 m^2^ g^−1^, which is larger than that of NW-MTS (12.035 m^2^ g^−1^). As the specific surface area of the electrode material increases, the number of available electroactive sites rises, resulting in more efficient transport channels for ions and charges within the material. Additionally, the pore size distribution and total pore volume for the two samples were calculated using the BJH model, as shown in Fig. 5b and d. The average pore diameters of NC-MTS and NW-MTS were 1.22 and 1.66 nm, respectively, exhibiting smaller pore size for NC-MTS, and also total pore volume of NC-MTS measured to be 0.036 cm^3^ g^−1^ while for NW-MTS, the values were calculated to 0.025 cm^3^ g^−1^.

**Figure 6:**
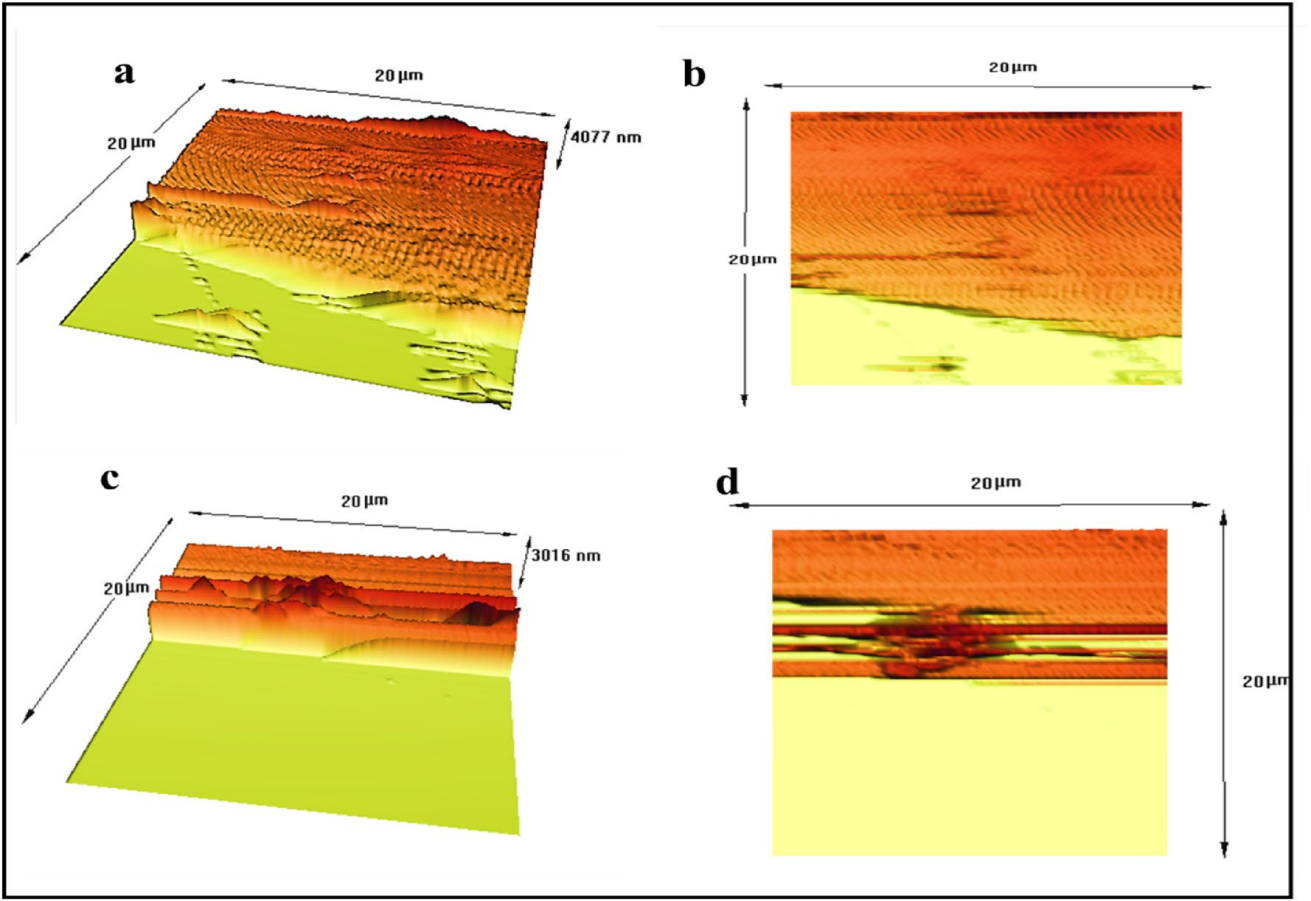
AFM images of NC-MTS (**a**, **b**) and NW-MTS (**c**, **d**).

To investigate three-dimensional surface morphology, roughness and thickness of NC-MTS/NF and NW-MTS/NF was taken by AFM images. Figure 6a and c display thicknesses of NC-MTS/NF and NW-MTS/NF as 4077 nm and 3016 nm, respectively. The surface morphology and roughness of the NW-MTS/NF were assessed using AFM, with a 20 × 20 µm area being examined. The surface of NW-MTS/NF was observed to be relatively smooth, with some regions showing randomly oriented accumulations of low and high peaks. According to the results, surface roughness values of NC-MTS/NF and NW-MTS/NF were found to be 589.73 nm and 123.00 nm, respectively (Fig. 6b and d).

### Electrochemical characterization of Mn_2_SnS_3_/NF electrode

**Figure 7:**
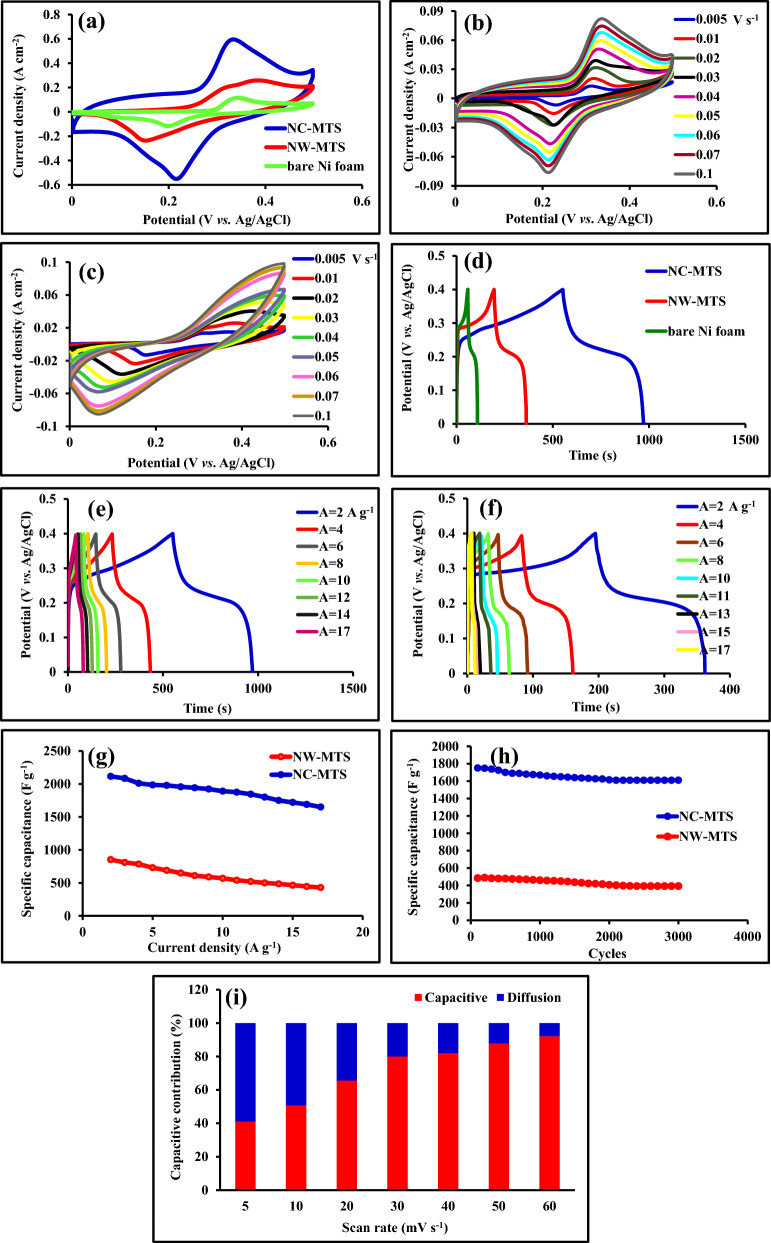
The CV curves of the bare Ni foam, NC-MTS/NF, and NW-MTS/NF at a scan rate of 30 mV s^−1^ (**a**); The CV curves of NC-MTS/NF electrode (**b**) and NW-MTS/NF (**c**) at various sweep rates; The GCD curves of the bare Ni foam, NC-MTS/NF and NW-MTS/NF at the current density of 2 A g^−1^ (**d**); The GCD curves of NC-MTS/NF electrode (**e**) and NW-MTS/NF electrode (**f**) at different current densities; Geometric specific capacitance of NC-MTS/NF electrode and NW-MTS/NF electrode at different current densities; (**g**) Cycling stability performance of NC-MTS/NF electrode and NW-MTS/NF at the current density of 9 A g^−1^ for 3000 cycles (**h**); Percentages of diffusive and capacitive contributions for as-obtained NC-MTS at various scan rates (**i**).

The electrochemical analyses are characterized by an electrochemical workstation in a three-electrode system involving NC-MTS and NW-MTS as working electrodes, Ag/AgCl as the reference electrode and platinum as the counter electrode in 3 mol L^−1^ KOH aqueous solution as electrolyte. In order to contrast the electrochemical characteristics of the bare Ni foam, NC-MTS and NW-MTS electrodes, cyclic voltammetry (CV), galvanostatic charge/discharge (GCD) measurements, and electrical impedance spectroscopy (EIS) measurements were employed. Figure 7a shows the typical cyclic voltammograms of bare Ni foam, NC-MTS and NW-MTS electrodes at the scan rate of 30 mV s^−1^ from 0.0 to 0.5 V potential range. As can be seen, bare Ni foam shows a pair of very insignificant redox peaks. It is evident that in comparison to NW-MTS, the NC-MTS electrode possesses the largest surrounding area and a higher current intensity which improves the electrochemical performance. Moreover, the CV curve of the NC-MTS electrode depicts a couple of redox peaks, illustrating the charge storage mechanism significantly comes from the reversible Faradaic redox processes in the alkaline (KOH) electrolyte at the electroactive surface. To gain further insight into the electrochemical performance of the produced electrodes, CV tests were performed at varying scan rates. Figure 7b,c show the typical CV curves of the NC-MTS and NW-MTS electrodes at scan rates ranging from 5 to 100 mV s^−1^. The distinctive redox peaks of each CV curve within the potential range 0.0–0.5 V can be attributed to the interaction between the electrolyte ions and the materials. With an increase in the scan rate, the cathodic and anodic peaks shift towards more negative and positive potentials, respectively, indicating a correlation with the internal resistance of the electrode and the restriction of charge transfer kinetics^42,43^. The shape of the CV curves is not remarkably influenced by the increment of the scan rate, which demonstrates improved mass transportation and electron conduction in the host materials. The GCD measurements were carried out to further evaluate the electrochemical performance of the prepared electrodes in 3 mol L^−1^ KOH aqueous electrolyte in the potential range of 0.0–0.4 V (vs. Ag/AgCl) at a current density of 2 A g^−1^ as displayed in Fig. 7d. The shape of the charge–discharge curves demonstrates the property of pseudo-capacitance following the CV results. Also, from the GCD results, the curve of the NC-MTS electrode displays the longest discharge time (423 s) in comparison to NW-MTS electrode (170.6 s) at identical current densities, indicating the higher charge storage capacity of the NC-MTS electrode. Meanwhile, according to the Eq. (1) and the discharge data, the areal capacitance of NC-MTS was calculated to be 2115 F g^−1^, while the areal capacitance of NW-MTS was calculated to be 853 F g^−1^. The NC-MTS electrode represents much further electrochemical properties than NW-MTS electrode. This can be attributed to the larger surface area of the electrode, which provides more active sites and channels for the storage and transfer of ions and electrons. Figure 7e,f exhibit the GCD curves of NC-MTS and NW-MTS electrodes at different current densities varying from 2 to 17 A g^−1^. As the current density increases, the specific capacitance gradually decreases. At high current densities, the transport of various ions in the electrolyte is impeded by time constraints, leading to insufficient utilization of the active materials, with only the external active surface utilized for charge storage^44^. As evident from the discharge curve, a distinct discharge platform is apparent, which corresponds to the CV curves, signifying excellent pseudocapacitive behavior. The results show that NC-MTS exhibits better specific capacitance performance compared to NW-MTS. In Fig. 7g, the plot illustrates that the specific capacitance decreases with increasing current density, which is due to the saturation of the electrode surface caused by the heavy infiltration of ions into the electrode. Conversely, at lower current densities, the increase in specific capacitance can be attributed to the filling of active sites on the electrode by electrolyte ions. It should be noted that the NC-MTS electrode demonstrates higher areal capacitances than NW-MTS electrode, and retention of approximately 78% capacitance even with an increase in current densities from 2 to 17 A g^−1^. This serves as evidence of the superior rate capability exhibited by the NC-MTS electrode. Whereas, NW-MTS electrode maintains about 50% capacitance retention after current densities increase from 2 to 17 A g^−1^. More significantly, the specific capacitance of NC-MTS is much more than previous similar reported values, as shown in Table 1.

**Table 1:** Comparison of the electrochemical performance of Mn_2_SnS_3_ electrode with other electrodes reported in the literature.

Electrode materials	Synthesis rout	Specific capacitance at current density	References
ZnCo_2_S_4_ core–shell nanospheres	Solvothermal method	1045.3 F g^−1^ at 1 A g^−1^	^44^
CoMo_2_S_4_/3D SG nanocrystal	Hydrothermal method	1288.33 F g^−1^ at 1 A g^−1^	^45^
NiS@CoS core–shell	Hydrothermal and electrodeposition methods	1210 F g^−1^ at 1 A g^−1^	^46^
CoS porous nanoflake	Facile biomembrane support system	366 F g^−1^ at 1 A g^−1^	^47^
SnS_2_/MoS_2_ heterojunction	Hydrothermal method	466.6 F g^−1^ at 1 A g^−1^	^48^
Mn-doped ZnS based different nanostructures	Hydrothermal method	1905 F g^−1^ at 1 A g^−1^	^49^
Heterostructured CuS/Fe_2_O_3_	Precipitation method	921 F g^−1^ at 1 A g^−1^	^50^
Nanocubes Cu_2_SnS_3_	Solvothermal method	2115 F g^−1^ at 2 A g^−1^	This work

To ensure that electrochemical capacitors can be used reliably over an extended period of time, it is important to investigate their long-term cycling stability, which can be done by performing a large number of GCD measurements, such as 3000, at a current density of 8 A g^−1^. The cycling life performance of the NC-MTS electrode was found to be superior to that of the NW-MTS electrode, as demonstrated in Fig. 7h. Specifically, after undergoing 3000 continuous cycles, the NC-MTS electrode retained 92% of its initial capacitance, while the NW-MTS electrode only retained 81%. These results clearly highlight the excellent cycling stability of the NC-MTS electrode.

To further understand the capacitive and diffusive contribution proportions of charge storage in a three-electrode system, the cyclic trend diagrams of the Ni-Mn-S/NiMn-LDH electrode were analyzed at various scan rates (from 5 to 50 mV s^−1^) by Eqs. (5) and (6):

5 $${\text{i}}\left( \upsilon \right) \, = k_{{1}} \upsilon + k_{{2}} \upsilon^{{{1}/{2}}}$$

6 $${\text{i}}\left( \upsilon \right)/\upsilon^{{({1}/{2})}} = k_{{1}} \upsilon^{{{1}/{2}}} + k_{{2}}$$

The capacitive behavior and the diffusive charging processes are represented by *k*_1_*υ* and *k*_2_*υ*^1/2^, respectively. Also, we were able to extract the *k*_1_ (slope) and *k*_2_ (intercept) parameters from the data of CVs according to Eq. (6). As seen in Fig. 7i, the surface capacitive contribution gradually rises from 41 to 92.14% with an increasing scan rate, showing that the charge storage mechanism is predominantly diffusion-controlled at lower scan rates. Overall, the results demonstrate the high scan rate restricted the diffusion of the ions into the inner region of the materials and offered a more capacitive contribution during the electrochemical reaction kinetics.

**Figure 8:**
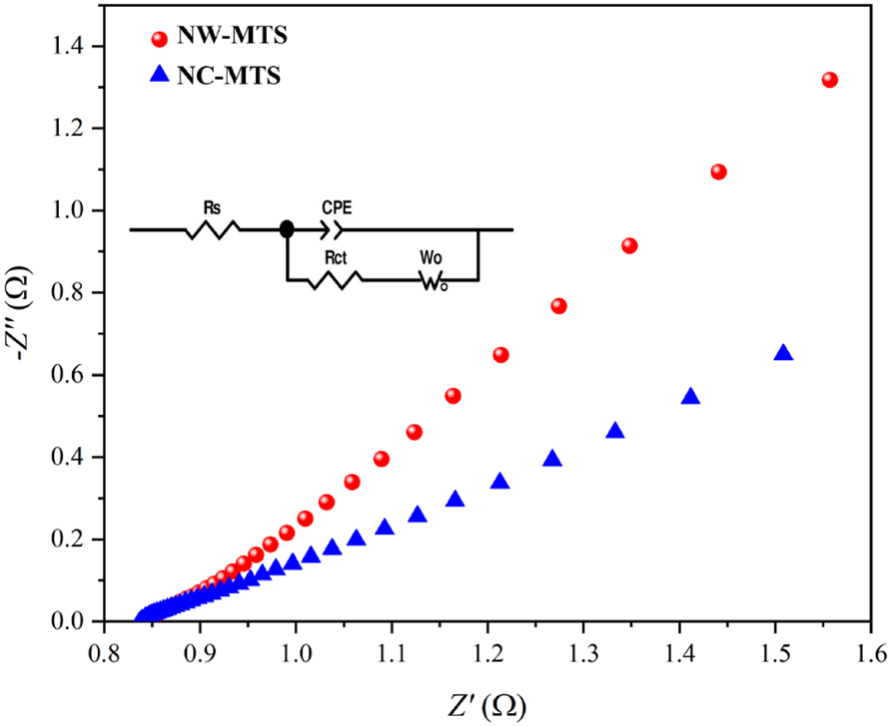
Impedance Nyquist plots of the NC-MTS/NF and NW-MTS/NF electrodes. The inset shows the corresponding equivalent circuit model used for the description of EIS.

To further investigate the electrical resistance response, electrochemical impedance spectroscopy (EIS) was conducted for both NC-MTS and NW-MTS electrodes. EIS study was employed at open circuit potential in the frequency range 0.01 Hz to 100 kHz in 3 mol L^−1^ KOH electrolyte. Typically, all of the EIS spectra's Nyquist plots consist of a semicircular part at the high-frequency region and a vertical straight line at the low-frequency region. The interface charge transfer resistance (*R*_ct_), which reflects the electron transfer kinetics at the electrode–electrolyte interface during the redox process, can be determined by analyzing the semicircle of the relatively high frequency band. In addition, the internal resistance of the electrochemical system (*R*_s_) can be obtained from the actual axis intercept in the high-frequency region, while the diffusion process is characterized by the linear part observed at lower frequencies. The superior electrochemical performance of the NC-MTS electrode, as compared to the NW-MTS electrode, is supported by the observations made in Fig. 8. The absence of a significant semicircle in the high-frequency region for the NC-MTS electrode suggests a much faster charge transfer rate, while the more vertical line observed in the low-frequency region indicates lower mass transfer resistance (Warburg). These findings are consistent with the specific capacity results and further underscore the superior electrochemical performance of the NC-MTS electrode. The equivalent circuit diagram simulated by the software Zview is shown in the inset of Fig. 8. According to the impedance fitting algorithm, the series resistance (*R*_s_) of NC-MTS is about 0.82 Ω and NW-MTS has achieved to be 0.85 Ω as depicted in Fig. 8. The *R*_ct_ value for NC-MTS is nearly 0.149 Ω, which is lower than the NW-MTS (1.435 Ω). As a result, The MTS electrodes exhibit a low series resistance and a nearly vertical line in the lower frequency range, indicating their potential as an alternative electrode material for supercapacitors, as evidenced by the results of this study.

### Electrochemical characterization of NC-MTS/NF//AC asymmetric device

**Figure 9:**
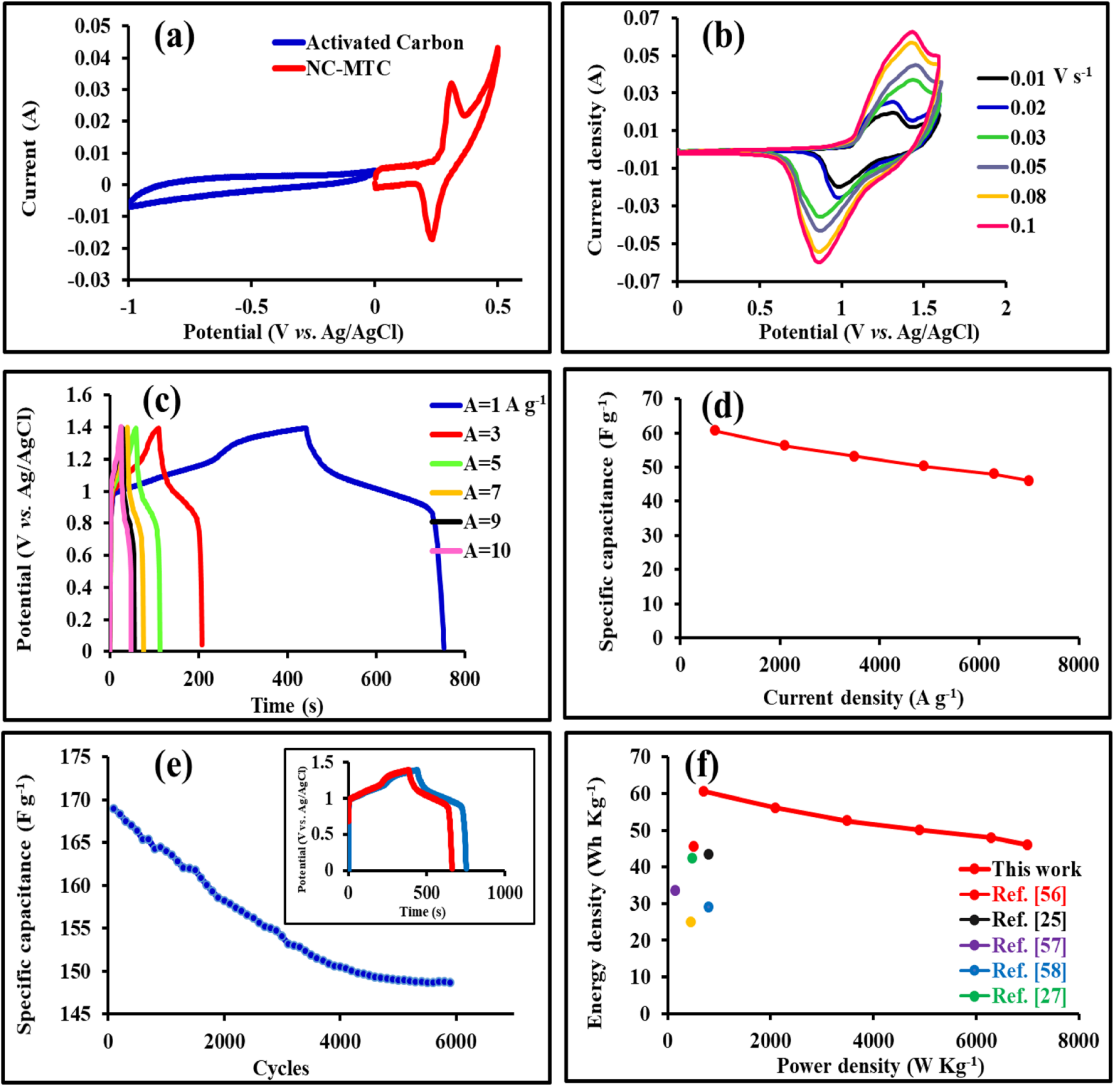
(**a**) CV curves of the NC-MTS/NF and AC electrodes at a scan rate of 20 mV s^−1^ in a three-electrode system; (**b**) CV curves of the asymmetric supercapacitor measured at different scan rates; (**c**) Galvanostatic charge–discharge curves at different current densities; (**d**) The specific capacitance of ASC device at various current densities; (**e**) Cycling stability of NC-MTS/NF//AC ACS electrode at the current density of 4 A g^−1^ for 6000 cycles (the inset shows the charge–discharge curves of the first and last cycles of the electrode); (**f**) The Ragone plot of the device at different current densities compared to previously reported asymmetric devices.

To demonstrate the potential utility of NC-MTS electrode arrays on Ni foam in high-performance electrochemical capacitors, an asymmetric device was constructed with NC-MTS serving as the positive electrode, activated carbon as the negative electrode, and 3 mol L^−1^ KOH as the aqueous electrolyte. Before constructing the two-electrode system, the CV curves were evaluated for both electrodes at a constant scan rate of 20 mV s^−1^ in the 3 mol L^−1^ KOH electrolyte. It can be concluded from the CV curves that the AC electrode operates within a potential range from  − 1.0 to 0.0 V, whereas the NC-MTS/NF electrode operates from 0 to 0.5 V (Fig. 9a). Thus, the fabricated asymmetric device can operate within 0 to 1.5 V. Hence, a potential window of 0.0–1.5 V was selected for further exploration of the electrochemical behavior of the ASC. Figure 9b shows the CV curves of the fabricated asymmetric supercapacitor at different scan rates from 10 to 100 mV s^−1^ within the potential window of 0.0 to 1.5 V. As demonstrated in the figure, the electrode nearly maintains its shape at a high scan rate of 80 mV s^−1^. Therefore, it can be concluded that the shape of CV curves does not change significantly as the scan rate increases. Figure 9c illustrates the GCD curves of the ASC device at various current densities from 1 to 10 A g^−1^ with a voltage window of 0 to 1.5 V as well. It could be observed that all the discharge curves were almost symmetric with their corresponding charging counterparts, indicating good electrochemical reversibility. The ASC exhibits an outstanding specific capacitance of 222.5 F g^−1^ at 1 A g^−1^, and still, it preserves a specific capacitance of 169 F g^−1^ at 10 A g^−1^. When operating at low current densities, the electrode's active sites can effectively interact with ions in the electrolyte, but at high charge–discharge rates, the specific capacitance decreases due to limited ion diffusion, causing the redox reaction to only take place on the surface of the active materials.

In addition to, the specific capacitances at various current densities were determined by analyzing the corresponding GCD curves of the NC-MTS//AC ASC, and the outcomes are depicted in Fig. 9d. The ASC preserves about 76% capacitance retention after current densities increase from 1 to 10 A g^−1^. The special high-rate performance of the device is ascribed to the distinctive structure of the active materials in the electrode, which enhances electrolyte transport across the electrodes. In order to evaluate the cyclic stability of the asymmetric supercapacitor, the device's GCD profile was monitored over 6000 cycles as shown in Fig. 9e. The device with 88% capacitance retention over 6000 cycles, exhibits excellent stability, surpassing many existing supercapacitors and demonstrating an impressive lifespan for the NC-MTS//AC ASC.

To assess the overall performance of the NC-MTS//AC asymmetric supercapacitor, compared to previously reported devices, the specific energy and specific power were calculated as shown in the Ragone plot (energy density *vs.* power density) in Fig. 9f. The power density and energy density are important parameters to investigate the performance of an energy storage device. The specific energy density and power density were calculated according to Eqs. (2) and (3), respectively. Encouragingly, the ASC displays a high energy density of 60.56 Wh kg^−1^, corresponding to a power density of 699.89 W kg^−1^, which is comparable to some of the reported devices, such as NiCo_2_S_4_//AC (45.5 Wh kg^−1^ at 512 W kg^−1^)^51^, Ni-Co-S/G//AC (43.3 Wh kg^−1^ at 800 W kg^−1^)^22^, NiCo_2_S_4_/Co_9_S_8_//AC (33.5 Wh kg^−1^ at 150 W kg^−1^)^52^, CoS/graphene//AC (29 Wh kg^−1^ at 800 W kg^−1^)^53^, NiCo_2_S_4_//G/CS (42.3 Wh kg^−1^ at 476 W kg^−1^)^26^, and Ni-Co sulfide//AC (25 Wh kg^−1^ at 447 W kg^−1^)^54^.

**Figure 10:**
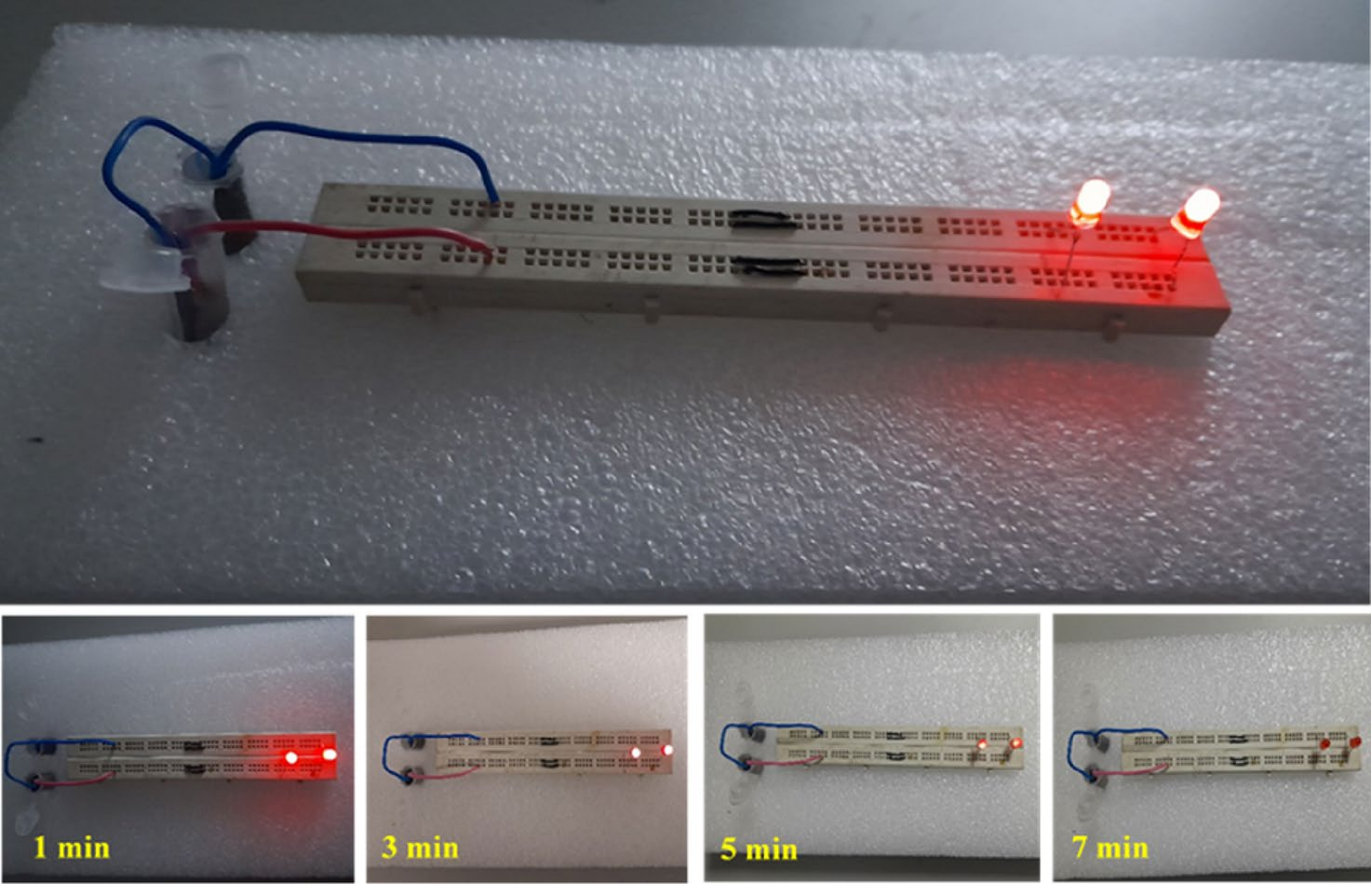
The brightness intensity of the LED indicator lit up by two ASCs connected in series for various time periods.

Eventually, to demonstrate the practical application of the prototype device, we attempted to power two red LEDs, which were successfully illuminated for 7 min by efficiently connecting two cells of the ASC device in series in a 3 mol L^−1^ KOH aqueous electrolyte (Fig. 10). The outcomes of this study substantiate that the NC-MTS//AC ASC device is a highly promising active material for practical energy storage applications.

## Conclusions

In summary, two types of MTS with distinct morphologies were synthesized using a solvothermal method. The NC-MTS electrode exhibited excellent pseudocapacitive performance, with a high specific capacitance of 2115 F g^−1^ at 2 A g^−1^, and exceptional cycle stability, with only an 8% capacitance loss after 3000 cycles. This is due to its good electrical conductivity, making it a promising candidate for use in energy storage devices. Additionally, an asymmetric supercapacitor was successfully fabricated using NC-MTS nanostructures and AC as positive and negative electrodes, respectively. The resulting device delivered a high specific energy of 60.56 Wh kg^−1^ at a specific power of 699.89 W kg^−1^. Most importantly, the ability of two ASCs connected in series to power two red LEDs indicates that the synthesized NC-MTS electrode material has significant potential for practical applications.

## Data Availability

The data that support the findings of this study are available within the article and its supplementary material.
